# Sex-Specific Temporal Patterns of Haemoglobin and Haematocrit as Mortality Predictors in Polytrauma Patients: A Watson Trauma Pathway Explorer Analysis

**DOI:** 10.3390/jcm15156076

**Published:** 2026-08-04

**Authors:** Lea Gröbli, Philipp Vetter, Cédric Niggli, Hans-Christoph Pape, Ladislav Mica

**Affiliations:** Department of Trauma Surgery, University Hospital of Zurich, 8091 Zurich, Switzerland

**Keywords:** polytrauma, haemoglobin, haematocrit, sex differences, mortality prediction, trauma resuscitation, early mortality, temporal patterns

## Abstract

**Introduction:** In polytrauma patients, effective triaging and resource allocation are critical in managing haemorrhage. This study aimed to analyse the association of haematocrit and haemoglobin with early death within 72 h in polytrauma patients stratified by sex. A sample of 3059 polytrauma patients was analysed using SPSS and the Watson Trauma Pathway Explorer up to 48 h after admission. **Methods:** A retrospective cohort of 3059 polytrauma patients (ISS ≥ 16) admitted 1996–2022 was analysed. Haemoglobin and haematocrit were measured at admission and serially through 48 h. Receiver operating characteristic (ROC) analysis assessed discriminatory performance for early death within 72 h. Multivariable logistic regression adjusted for injury severity, age, physiological status, and resuscitation volumes. **Results:** Significant sex-based differences in haemoglobin and haematocrit were observed at all time points (*p* < 0.05) in univariable analysis; haemoglobin and haematocrit showed significant associations with mortality in males at multiple time points (admission, 1 h, 3 h, 12 h, 24 h) and in females at admission and 1 h. After multivariable adjustment, haematocrit and haemoglobin for males at 2 h remained significant. Discriminatory performance was poor across all time points: in males, haemoglobin AUC ranged from 0.51 (95% CI 0.33–0.68) at admission to 0.59 (95% CI 0.43–0.75) at 48 h; in females, from 0.44 (95% CI 0.16–0.72) at 1 h to 0.68 (95% CI 0.44–0.93) at 3 h. Females exhibited a higher mortality in the early hours and at 8 h after admission. Mortality thresholds were lower in females than males at 1 h,3 h, 6 h, 24 h and 48 h for haematocrit and at 1 h, 2 h, 3 h and 8 h for haemoglobin, with the lowest value at 2 h for haemoglobin (females: 7.55 g/dL, males: 8.5 g/dL) and 1 h for haematocrit in females. **Conclusions:** While crude comparisons demonstrated sex-specific temporal patterns in haemoglobin and haematocrit between survivors and non-survivors, these associations did not remain statistically significant after multivariable adjustment and Benjamini-Hochberg correction for multiple testing (all FDR-adjusted *p* ≥ 0.200). Associations were substantially confounded by resuscitation practices. Discriminatory performance was poor across all time points (AUC 0.44–0.68). These findings should be considered hypothesis-generating and require prospective validation in cohorts with standardised resuscitation protocols before clinical implementation.

## 1. Introduction

Polytrauma patients need complex and timed management to ensure optimal outcomes, particularly in cases involving exsanguination, a leading cause of mortality in the early hours post-trauma [1,2]. Immediate surgical intervention to control life-threatening haemorrhage, alongside the treatment of coagulopathy of trauma shock, is essential [3,4]. Haematocrit and haemoglobin levels are critical indicators of physiological status and prognostic outcomes. Females typically present with lower haemoglobin and haematocrit levels compared to males; however, the impact of these differences on survival outcomes remains unclear [5,6,7,8,9].

This study was designed to investigate the relationship between haemoglobin and haematocrit levels and early death within 72 h in polytrauma patients, with an emphasis on sex-based differences. We aim to determine whether critical threshold values for these parameters can be identified at various time points post-admission, which could influence clinical decision-making, particularly in the allocation of blood products and fluid resuscitation [10]. The initial blood volume management was practised according to ATLS (Advanced Trauma Life Support) principles [11], though resuscitation strategies evolved substantially during the study period with the adoption of damage control resuscitation protocols [12]. Previous research has demonstrated a predictive value for haemoglobin and haematocrit upon admission in relation to 7-day in-hospital mortality [13]. Additional studies highlight the correlation between low haemoglobin levels, PT (prothrombin time), aPTT (activated partial thromboplastin time) and PLT (platelet count) and poor outcomes. This correlation was shown to be stronger for more severely injured patients and explains the correlation of haemoglobin level (Hb) and severe injuries [14]. However, the predictive utility of these markers over time and their potential for improving mortality prediction remains under-explored. The Watson Trauma Pathway Explorer^®^ (Zuerich, Switzerland) is an outcome AI-based prediction tool that can predict adverse events (AE) such as sepsis, SIRS and early death in 72 h, using an internal database of more than 3500 patients with ongoing inclusion [15]. This allowed us to compare individual parameters of patients to a reference group up until 21 days after admission.

We hypothesised that haemoglobin and haematocrit would demonstrate time-dependent associations with early death within 72 h in polytrauma patients; these associations would differ between male and female patients due to baseline haematological differences and sex-specific physiological responses to haemorrhage, and critical threshold values could be identified at specific time points to inform resuscitation strategies.

## 2. Methods

### 2.1. Patient Sample

This retrospective cohort study included 3059 polytrauma patients, aged 16 years and above, with an Injury Severity Score (ISS) of 16 or higher, admitted to the trauma bay of the University Hospital of Zurich. Patients admitted between 1996, when the recording of this database began, and 2022 were considered in this study. The ISS cutoff was chosen, as this is the most widely agreed-upon definition of what constitutes a polytraumatised patient [16]. Of these, 2250 were male, and 809 were female. Not all patients showed values for all time points, as some were in surgery during the time points or died and therefore did not have any other values after the time point of death. To provide a good analysis regarding early death within 72 h, these patients were also included. Exclusion criteria included patients who died prior to admission and those transferred from other hospitals. Data for haemoglobin and haematocrit levels were collected at multiple time points: admission, 1 h, 2 h, 3 h, 4 h, 6 h, 8 h, 12 h, 24 h, and 48 h after admission to our trauma bay. The primary outcome was early death within 72 h of admission, defined as death occurring between hospital arrival and 72 h post-admission, regardless of location (emergency department, operating room, or intensive care unit).

### 2.2. Laboratory Analysis

All laboratory values were measured by the Institut für klinische Chemie, University of Zurich. Haematocrit was quantified as a percentage (%) and haemoglobin in grams per decilitre (g/dL). The analysis adhered to standard laboratory protocols, with routine calibration and validation of equipment.

### 2.3. Statistical Analysis

The data were analysed using SPSS version 29.0 (IBM, Armonk, NY, USA), as well as the Watson Trauma Pathway Explorer^®^ (© by IBM, Switzerland and L. Mica, Zuerich, Switzerland). Males and females were compared in terms of haemoglobin and haematocrit, as well as for early death within 72 h in males and females, in four further sub-groups. Numerical Values were analysed using the number of patients, as well as the mean and its standard deviation (SD). Ordinal data were analysed using the median and interquartile range (IQR). Binary data were analysed using percentages. Descriptive statistics were computed, and group differences were assessed using the unpaired t-test (numerical data) and Mann-Whitney U test (differences in two unrelated groups). Univariable and multivariable binary logistic regression were performed to assess whether haemoglobin and haematocrit independently predicted early death within 72 h. Univariable models included only haemoglobin or haematocrit as the predictor variable to assess crude associations with mortality. Multivariable models adjusted for potential confounders including Injury Severity Score (ISS), age, APACHE II score at admission, cumulative fluid volume administered, and cumulative erythrocyte concentrate units transfused up to each time point. These covariates were selected a priori based on established associations with trauma mortality and their potential to confound the relationship between haematological parameters and outcomes [10,16,17,18,19,20,21]. Both crude (univariable) and adjusted (multivariable) odds ratios with 95% confidence intervals were calculated. Both crude (univariable) and adjusted (multivariable) odds ratios with 95% confidence intervals were calculated. Model fit was assessed using the Nagelkerke R^2^ statistic.

Receiver operating characteristic (ROC) curves were constructed for haemoglobin and haematocrit at each time point, with early death within 72 h as the outcome. The area under the curve (AUC) with 95% confidence intervals was calculated to quantify discriminatory performance. AUC values were interpreted as: 0.50–0.60 (no discrimination), 0.60–0.70 (poor), 0.70–0.80 (acceptable), 0.80–0.90 (excellent), >0.90 (outstanding) [21,22,23].

To account for multiple comparisons across 40 statistical tests (haemoglobin and haematocrit at 10 time points in 2 sex groups), the Benjamini-Hochberg false discovery rate (FDR) correction was applied to *p*-values from Mann-Whitney U tests and logistic regression analyses. Adjusted *p*-values < 0.05 were considered statistically significant after correction [24].

Optimal threshold values were determined using the Euclidean distance method (also known as the “closest-to-top-left” method), which identifies the point on the ROC curve geometrically closest to perfect classification (sensitivity = 1, specificity = 1) [25]. For each potential cutoff value, the Euclidean distance was calculated as:

$$\mathrm{Distance}=\sqrt{{\left(1-\mathrm{Sensitivity}{)}^{2}+(1-\mathrm{Specificity}\right)}^{2}}$$

The cutoff value with the minimum distance was selected as the optimal threshold [25]. This method efficiently identifies the point closest to the top-left corner of ROC space and provides balanced sensitivity and specificity when both are valued equally [25]. For each optimal threshold, sensitivity, specificity, positive predictive value, and negative predictive value were calculated. All ROC analyses were performed separately for male and female patients.

**Missing data:** Not all patients had laboratory measurements at all time points due to ongoing surgical procedures, early death, or discharge from the trauma bay. The number of available measurements at each time point is reported in Tables 2 and 5. Patients who died prior to a given time point were excluded from analyses at subsequent time points but were included in all analyses up to their time of death to avoid survivor bias. No imputation was performed for missing laboratory values.

**Sensitivity analyses for data quality:** Visual inspection and statistical outlier detection identified erroneous data entries at specific time points. The values which were clearly entered incorrectly due to these values being impossible to attain were removed, and the analysis was rerun.

### 2.4. Ethical Approval

This study was conducted according to good clinical practices, in accordance with the Helsinki Declaration. The Analysis of the data of these patients was approved by the ethical commission of the University Hospital of Zurich, as well as the government of Zurich upon development of the database (Nr. StV 1-2008) and reapproved for the development of the Watson Trauma Pathway Explorer^®^ (BASEC 2021-00391). The research was based on the TRIPOD statement, representing a guideline for multivariable prediction models [26].

## 3. Results

### 3.1. Patient Sample

**Figure 1:**
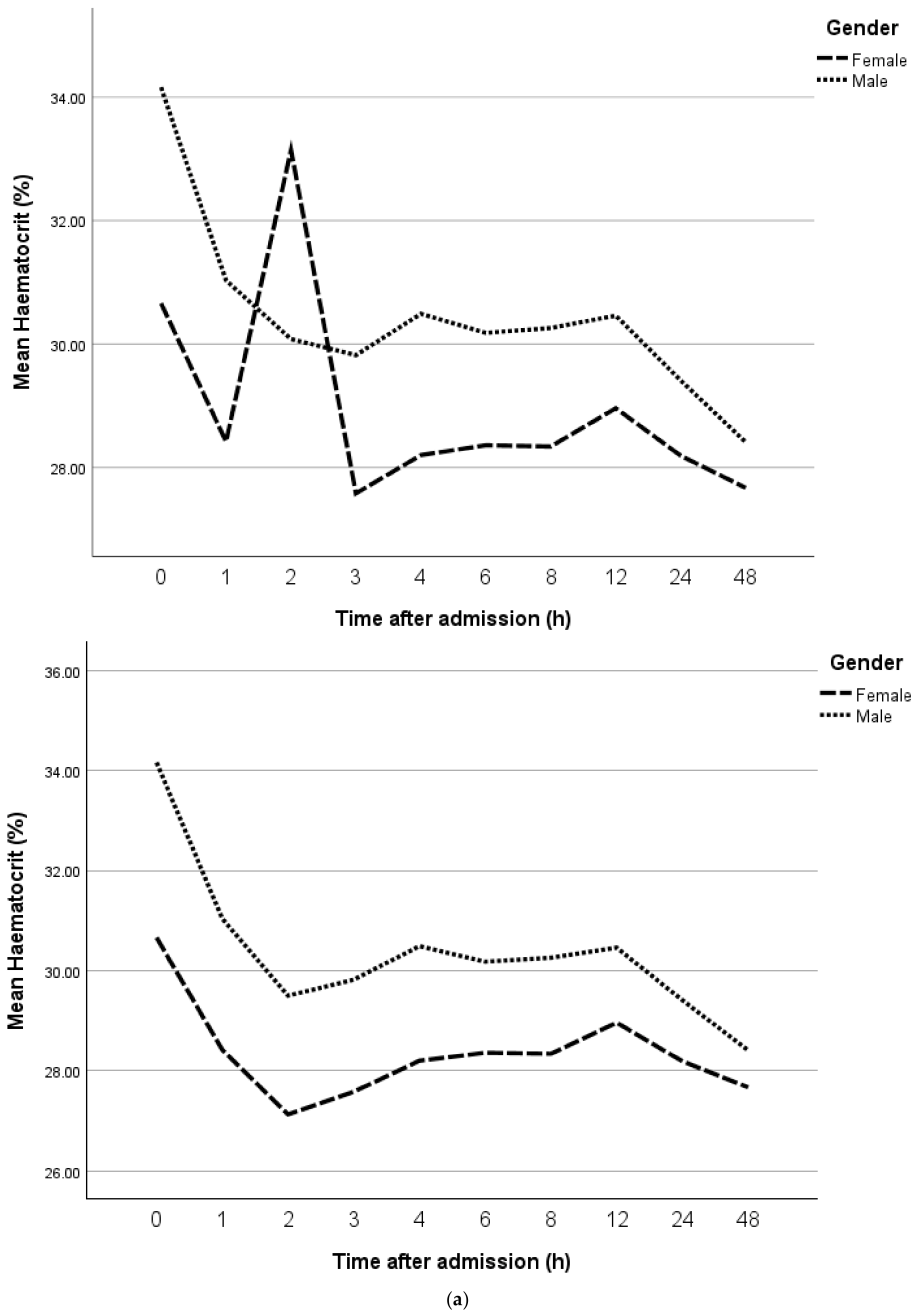

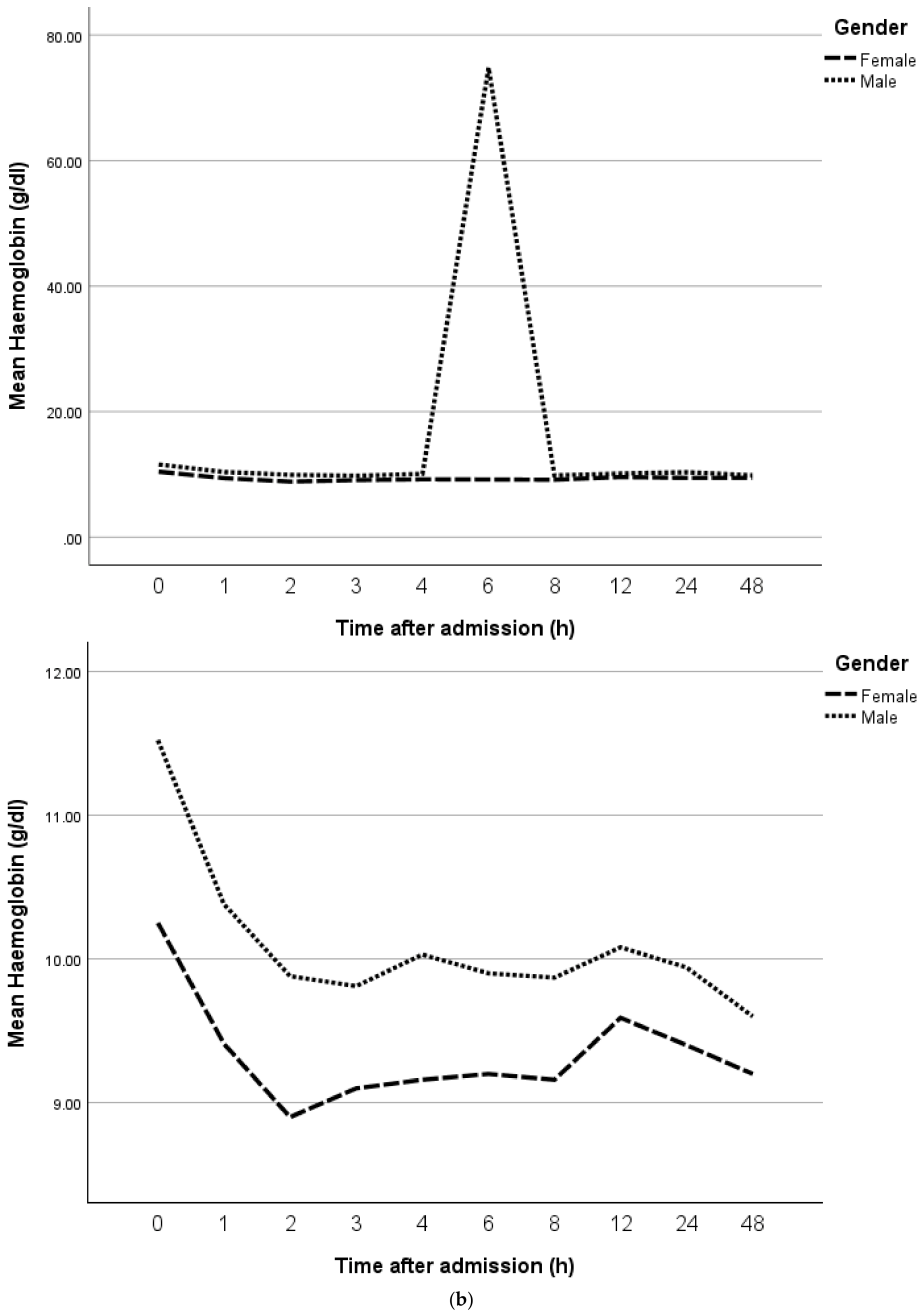
(**a**): Haematocrit (%) by sex over 48 h, before implausible values were removed and after implausible values were removed. All differences *p* < 0.05. (**b**): Haemoglobin (g/dL) by sex over 48 h, before implausible values were removed and after implausible values were removed. All differences *p* < 0.05.

The study sample consisted of 3059 polytrauma patients with a mean age of 45.81 years (SD ± 20.13), and 73.55% of the cohort were male (*n* = 2250), while 26.45% were female (*n* = 809). The cohort was characterised by varying mortality outcomes, with a higher proportion of older patients, a significantly higher Injury Severity Score (ISS) [9], NISS (New Injury Severity Score) [27], and Acute Physiology and Health Evaluation II (APACHE II) [11] scores in those who did not survive. The Glasgow Coma Scale (GCS) [28] score at admission was notably lower in the non-survivors (mean of 3) compared to survivors (mean of 13). Additionally, non-survivors had a significantly lower pH and higher lactate levels. All of which were statistically significant (*p* < 0.05) (Table 1). Table 2 shows the distribution of the haemoglobin and haematocrit values measured in the population as well as their median and IQR (interquartile range). The Data outliers at 6 h for haemoglobin in males that can be seen in Table 2 and Figure 1b are due to data points that have been entered into the database incorrectly, but we did not want to omit these data points so as not to lose the rest of the values.

The proportion of patients with available laboratory measurements decreased over time, from 88.8% (*n* = 2716/3059) at admission to 47.5% (*n* = 1452/3059) at 48 h. Patients with missing measurements at later time points had higher injury severity (median ISS 34 vs. 26, *p* < 0.001), lower admission GCS (median 3 vs. 10, *p* < 0.001), and higher mortality (42.3% vs. 18.7%, *p* < 0.001) compared to those with complete data, indicating missing-not-at-random mechanisms.

### 3.2. Haemoglobin and Haematocrit Analysis

At baseline and across subsequent time points, both haemoglobin and haematocrit levels showed a tendency to decrease over time. This trend was consistent across both male and female patients. Upon comparing male and female patients, females consistently had lower haemoglobin and haematocrit levels compared to their male counterparts. (Figure 1) All values in Figure 1 showed a significant *p*-value.

**Figure 2:**
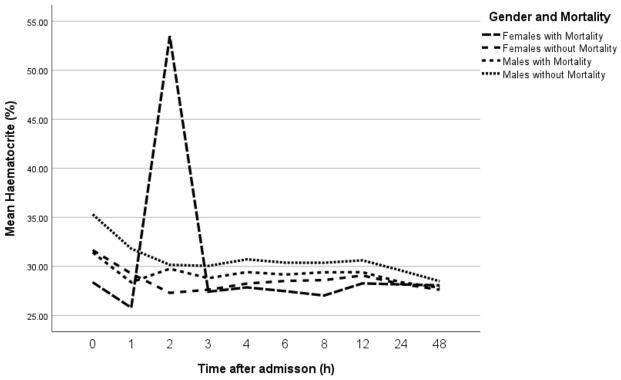

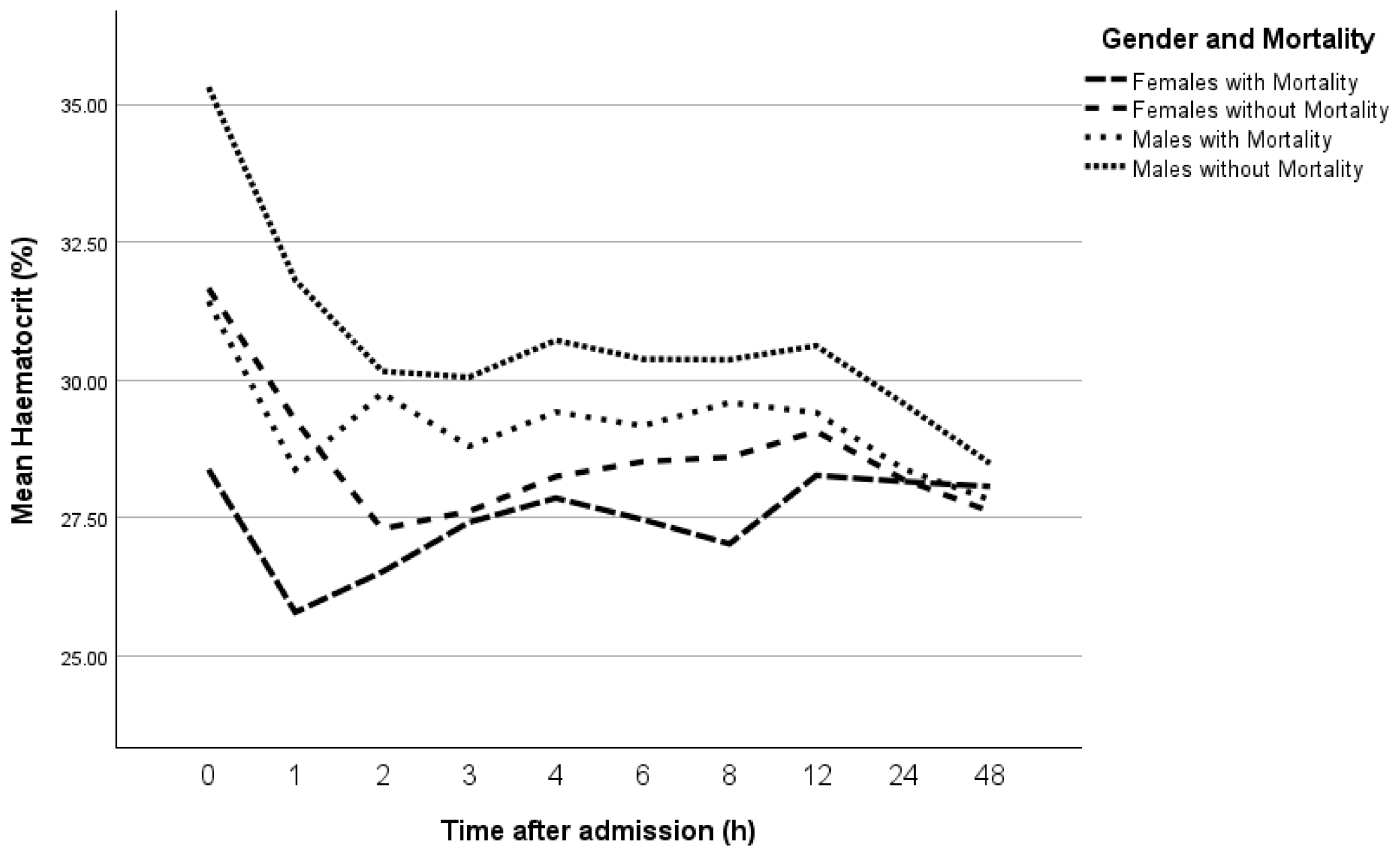
Haematocrit (%) trajectories by sex and mortality status. Before implausible values were removed and after implausible values were removed.

**Figure 3:**
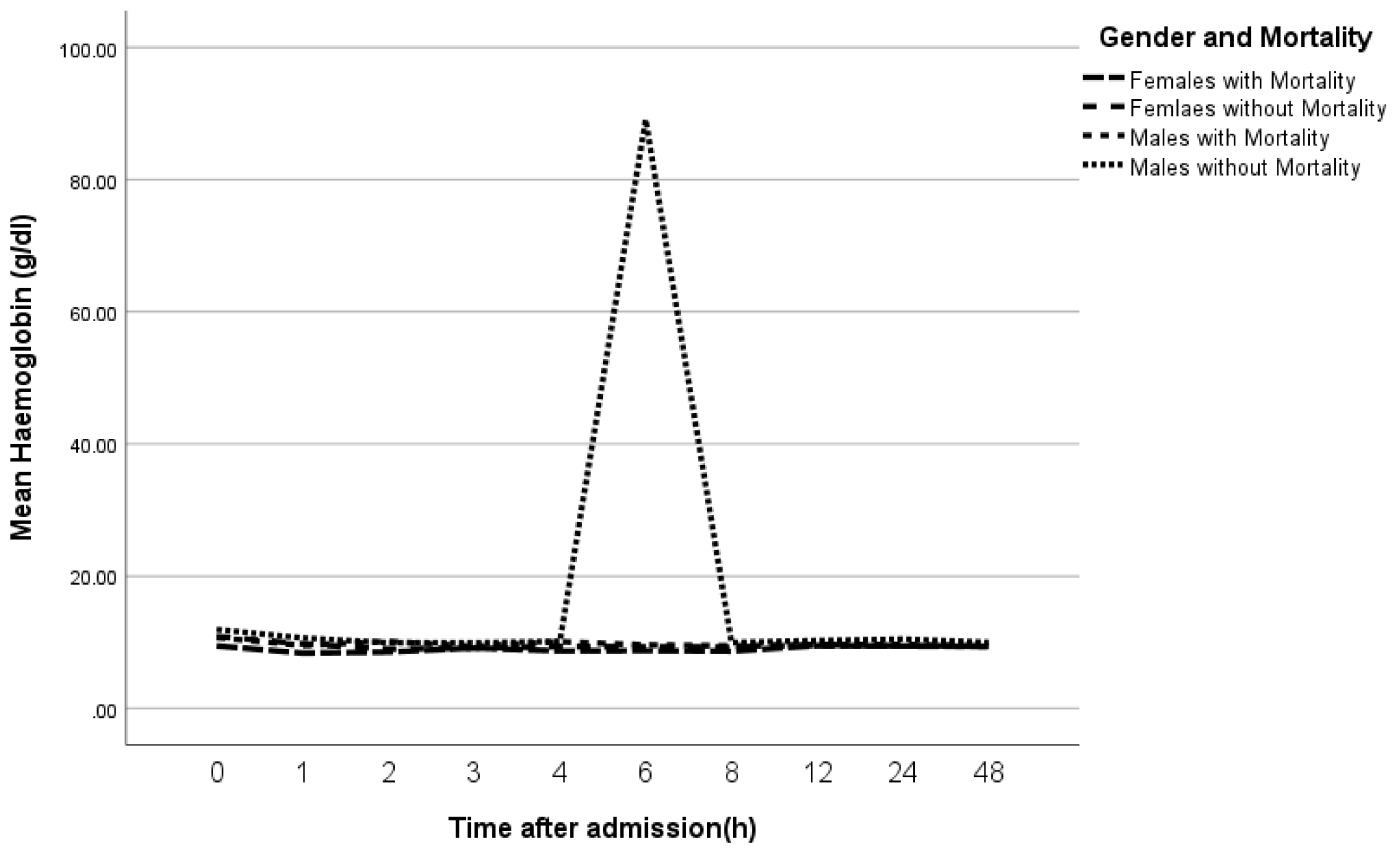

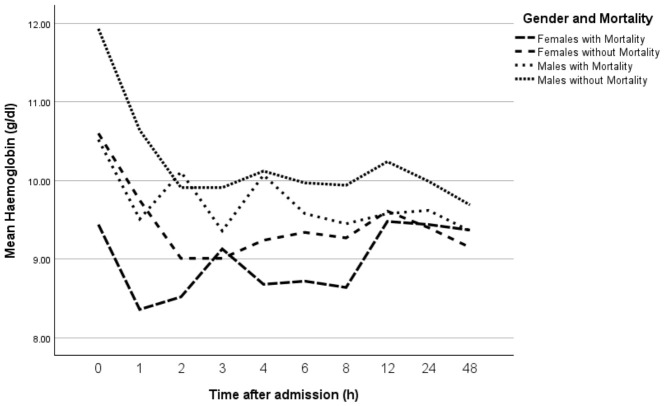
Haemoglobin (g/dL) trajectories by sex and mortality status over 48 h. Before implausible values were removed and after implausible values were removed.

Following the analysis above, we split the cohort into males and females to analyse their mortality for the haematocrit and haemoglobin at the above-mentioned points in time. As in the combined group, the male group that survived also had a higher haematocrit and haemoglobin than the group that passed away. The statistical analysis revealed significant differences in haemoglobin and haematocrit levels between survivors and non-survivors across various time points. For the male cohort, the haematocrit at admission, 1 h, 12 h, and 24 h (Figure 2), as well as haemoglobin at admission, 1 h, 3 h, 12 h, and 24 h, showed significant associations with early death within 72 h (*p* < 0.05) (Figure 3). For the female population, the surviving population also had a higher haematocrit and haemoglobin. For females, both haemoglobin and haematocrit were significant at admission and 1 h post-admission, with lower levels correlating to more early death within 72 h in these time frames (*p* < 0.05) (Figure 2 and Figure 3). When comparing the male and female values for both haemoglobin and haematocrit, the female population constantly had lower values.

Visual inspection identified 2 outlier values for male haemoglobin at the 6-h time point (0.1 and 42,015 g/dL, compared to the cohort median of 9.5++ g/dL after outlier removal). In the primary analysis excluding these outliers, male haemoglobin at 6 h showed a significant association with early death within 72 h (crude OR 1.85, 95% CI 1.07–3.22, *p* = 0.029), though this did not persist after multivariable adjustment (*p* = 0.873). At the 2-h time point, 1 outlier values for female haematocrit were identified (1537%, compared to the cohort median of 28.5%). In the primary analysis excluding these outliers, female haematocrit at 2 h showed a non-significant association with early death within 72 h (*p* = 0.574). This suggests the finding is unreliable.

### 3.3. Volume and Erythrocyte Concentrate Analysis

The volume of fluids and erythrocyte concentrates administered was higher in male patients compared to females. However, despite the differences in transfusion volumes, the surviving patients had lower volumes and erythrocyte concentrate transfused. This indicates that while volume and erythrocyte concentrate administration were associated with survival, other factors likely influenced the outcomes, such as differences in haemoglobin and haematocrit levels (Table 3 and Table 4).

### 3.4. Univariable and Multivariable Regression Analysis

Binary logistic regression was performed to assess whether haemoglobin and haematocrit independently predicted early death within 72 h (Table 5). In univariable models, haemoglobin showed crude associations with mortality in males at 2 h (OR 2.32, 95% CI 1.19–4.53, *p* = 0.01) and 6 h (OR 1.85, 95% CI 1.07–3.22, *p* = 0.029). Haematocrit demonstrated crude associations in males at 2 h, 4 h, 6 h, 8 h, and 24 h (all *p* < 0.05). In females, only haematocrit at 48 h showed a significant crude association (OR 1.54, 95% CI 1.01–2.35, *p* = 0.045). Several models in the female subgroup failed to converge or produced unstable estimates due to small sample sizes and low event rates.

After adjusting for ISS, age, APACHE II, cumulative fluid volume, and cumulative erythrocyte concentrate transfusion in multivariable models, only haemoglobin (adjusted OR 1.13, 95% CI 1.03–1.24, *p* = 0.01) and haematocrit (adjusted OR 1.04, 95% CI 1.01–1.07, *p* = 0.009) at 2 h in males remained statistically significant. However, after Benjamini-Hochberg false discovery rate correction for 40 comparisons, none of these associations remained significant (all FDR-adjusted *p* ≥ 0.200) (Table 6).

Nagelkerke R^2^ values for multivariable models ranged from 0.11 to 0.39, with the highest values observed at admission and the lowest at 48 h, indicating that the combination of haemoglobin or haematocrit with established trauma severity markers explained 11–39% of the variance in early mortality.

### 3.5. Time-Related Threshold Values in Haemoglobin and Haematocrit Between Groups According to Early Death Within 72 h

**Figure 4:**
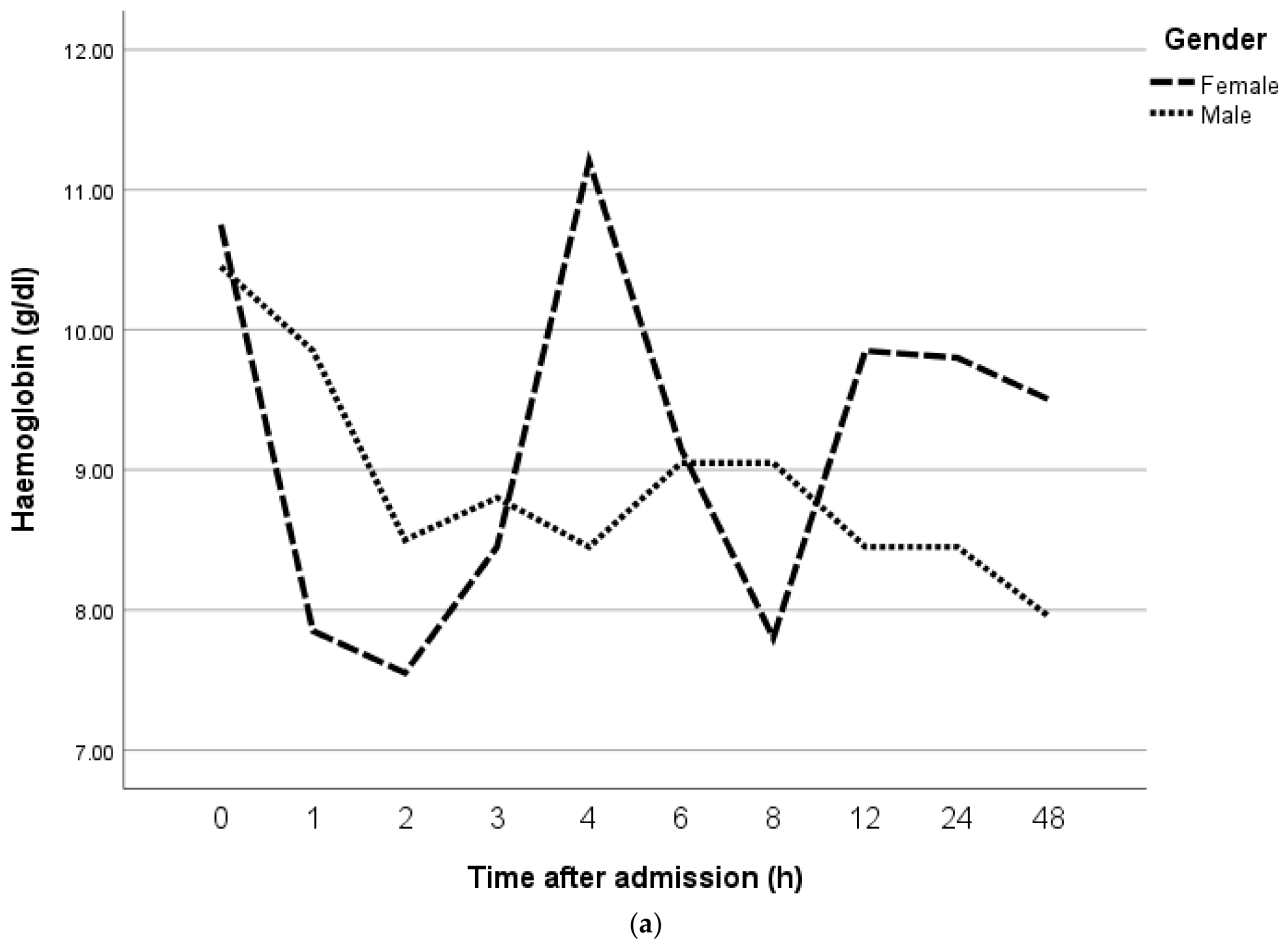

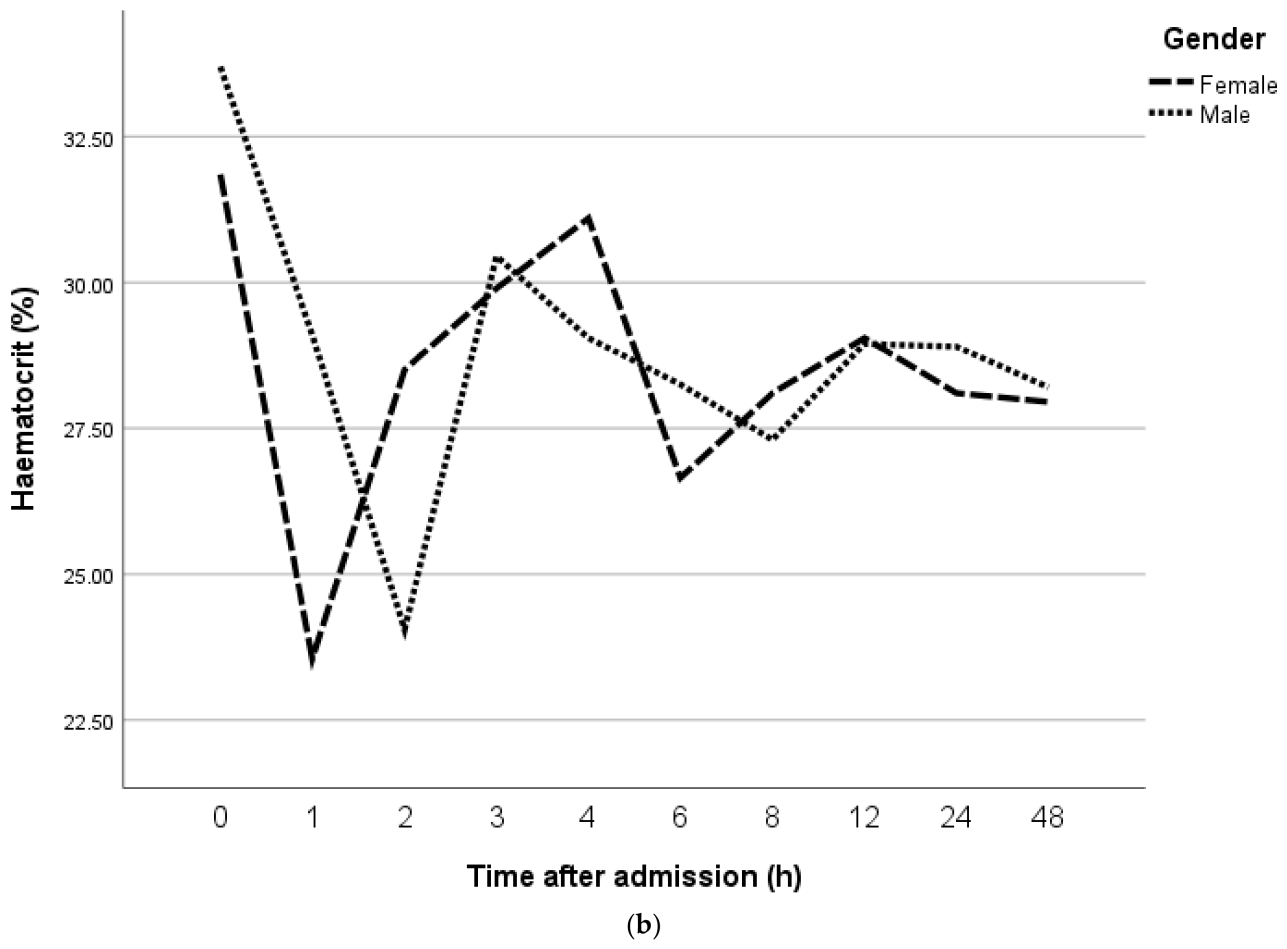
(**a**): Optimal haemoglobin (g/dL) thresholds for early death prediction by sex over 48 h. (**b**): Optimal haematocrit (%) thresholds for early death prediction by sex over 48 h.

We used a top-left method to calculate the threshold values. It indicates the maximum amount of combined sensitivity and specificity. The predictive quality of early death within 72 h prediction based on haemoglobin and haematocrit levels was determined for each time point. For haematocrit, the threshold value decreased until 1 h in females and 2 h in males (Figure 4a). For haemoglobin, the threshold values decreased until 2 h in females and 48 h in males (Figure 4b). These findings highlight the critical time points at which haemoglobin and haematocrit levels can serve as useful predictors of early death within 72 h. They tended to recover afterwards, however, without reaching the initial levels. Haematocrit becomes crucial after four hours, and regarding haemoglobin, it becomes crucial after two hours.

The discriminatory performance of haemoglobin and haematocrit for early death within 72 h prediction varied across time points. For haemoglobin in males, AUC values ranged from 0.51 (0.33–0.68) at admission to 0.59 (0.43–0.75) at 48 h. In females, AUC values ranged from 0.65 (0.44–0.86) at admission to 0.65 (0.39–0.01) at 48 h (Table 7). AUC values below 0.70 indicate limited discriminatory ability, while values above 0.80 suggest good discrimination. Time points with AUC < 0.70 should be interpreted with caution as clinically useful predictive thresholds.

## 4. Discussion

The use of haemoglobin and haematocrit as predictors of early death within 72 h in polytrauma patients provides insight into the cause of trauma-related death and bleeding [13,14]. A time-related analysis for haemoglobin and haematocrit and their association with early death (within 72 h) was performed using the IBM Watson Trauma Pathway Explorer^®^ [8].

Traditionally, trauma severity scores such as the ISS, NISS, and APACHE II are used to predict outcomes in polytrauma patients [10,16,18,19,20]. These scores focus on injury severity and physiological responses, but they do not always consider the patient’s baseline health status or the temporal changes in physiological markers such as haemoglobin and haematocrit. Today’s population is on average older and sicker than in earlier years [29]. In trauma patients, bleeding and acute blood loss are certainly one of the biggest risk factors for early death within 72 h [2,30].

Our study further highlighted significant sex-based differences in the predictive value of haemoglobin and haematocrit. Males exhibited a stronger association between these markers and early death within 72 h at multiple time points, while the predictive value in females was most significant in the first hour post-admission and at 48 h. This finding is in line with prior research that has suggested that females may have a different physiological response to trauma compared to males, potentially due to differences in baseline haemoglobin and haematocrit levels, hormonal influences, or other biological factors [5,6,7,8,9,31]. The lower haemoglobin and haematocrit levels observed in females, along with their higher mortality in the early hours after trauma, suggest that females may be at a disadvantage when it comes to survival, particularly if early interventions are not optimised.

The sex-specific haemoglobin and haematocrit differences observed in this study must be interpreted in the context of established baseline physiological variation. Healthy adult males demonstrate haemoglobin values approximately 1.5–2.5 g/dL higher than females (male reference range 13.7–19.2 g/dL vs. female 12.5–16.4 g/dL), driven primarily by testosterone-mediated erythropoiesis [5,6,7]. Sex-specific threshold differences varied across time points. At admission, females demonstrated slightly higher thresholds than males (10.75 g/dL vs. 10.45 g/dL), but at most subsequent time points, females had lower thresholds. The lowest threshold occurred at 2 h for haemoglobin (7.55 g/dL in females vs. 8.5 g/dL in males) and 1 h for haematocrit in females. These patterns, when compared to baseline sex differences in haemoglobin (males 1.5–2.5 g/dL higher than females), suggest that the sex-specific mortality thresholds we identified may reflect possible physiological responses to haemorrhage. The predominantly lower mortality thresholds observed in females (at 1 h, 2 h, 3 h, 8 h for haemoglobin and 1 h, 3 h, 6 h, 24 h, 48 h for haematocrit) must be interpreted cautiously. While these lower thresholds might suggest females tolerate lower haemoglobin/haematocrit values less well than males, this pattern likely reflects the combined effect of baseline physiological differences (females start with lower values), sex-specific resuscitation practices (females received less volume and fewer erythrocyte concentrates), and potentially different injury patterns or physiological reserve. The clinical implication is that applying uniform haemoglobin thresholds across sexes may fail to account for these multifactorial differences.

The temporal patterns of haemoglobin and haematocrit predictive value we observed—particularly the early predictive window in females (admission and 1 h) compared to the more distributed pattern in males (admission, 1 h, 3 h, 12 h, 24 h)—align with established sex dimorphisms in coagulation and haemorrhagic response. Females demonstrate baseline hypercoagulability compared to males, characterised by shorter clotting time, increased clot propagation, enhanced clot strength, and decreased fibrinolysis [32,33,34]. This hypercoagulable phenotype persists after traumatic injury and confers a survival benefit, with hormonally active females demonstrating 14% decreased odds of death compared to males when shock is superimposed on trauma [35]. Oestrogen appears to play a central mechanistic role: circulating oestradiol levels increase in correlation with injury severity, and experimental models demonstrate that oestrogen administration after trauma-haemorrhage improves cardiovascular function, reduces inflammatory cytokine production, and decreases mortality [35,36,37,38,39]. The early predictive significance of haemoglobin and haematocrit in females observed in our study may therefore reflect a critical window during which oestrogen-mediated protective mechanisms are most active. However, our inability to control for menstrual cycle phase, menopausal status, or exogenous hormone use (oral contraceptives, hormone replacement therapy) limits mechanistic interpretation of these sex-specific temporal patterns.

Sex-based differences in inflammatory response to polytrauma may further explain the temporal patterns we observed. Male trauma patients demonstrate more robust and prolonged proinflammatory cytokine production (IL-6, IL-8, IL-1β, TNF-α) compared to females, associated with higher rates of multiple organ failure, sepsis, and longer intensive care unit stays [40,41,42,43]. Conversely, females exhibit earlier upregulation of adaptive immunity markers and wound healing processes, suggesting accelerated recovery [44]. These inflammatory differences are modulated by both sex hormones and X-linked genetic polymorphisms [45]. The higher mortality we observed in females during the early hours (admission, 1 h) despite their baseline hypercoagulability may reflect a subset of female patients who, when injured severely enough to overcome their physiological protective mechanisms, experience disproportionately poor outcomes. This hypothesis is supported by recent evidence that post-menopausal females (≥45 years) demonstrate increased fibrinolysis and higher mortality compared to younger females and males of all ages, suggesting that hormonal status critically modulates trauma outcomes in females [46].

Interestingly, while both male and female patients who survived had higher haemoglobin and haematocrit levels compared to those who died, the amount of volume and erythrocyte concentrate transfused did not entirely explain the differences in survival outcomes. Although males received more volume and erythrocyte concentrates, females still exhibited lower haemoglobin and haematocrit levels, which suggests that factors beyond transfusion volumes may contribute to the differences in survival outcomes. This finding points to the possibility that the effectiveness of transfusions, or other unidentified variables such as metabolic or hormonal differences, may influence early death within 72 h in polytrauma patients, particularly females [47,48,49]. According to our data, the best timeframe to intervene is in the first two hours after a patient has been admitted to hospital. Afterwards, the window of opportunity closes again.

It is also important to note that while haemoglobin and haematocrit levels provide valuable prognostic information, they are not the sole determinants of early death within 72 h in polytrauma patients. Factors such as the extent of injuries, comorbidities, and pre-existing health conditions, which are not fully accounted for in this study, also play a significant role in patient outcomes [18,19,20,50,51]. Furthermore, blood loss and surgical interventions, which may alter haemoglobin and haematocrit levels, could have confounded our findings. The retrospective nature of this study limits the ability to account for all variables that might influence early death within 72 h, such as variations in surgical timing or internal bleeding.

The predictive performance of haemoglobin and haematocrit can be contextualised against published trauma literature. Lam et al. reported that admission haemoglobin demonstrated moderate discriminatory ability for 7-day mortality (AUC 0.734) in 1673 trauma patients, similar to our findings at 3 h for females for haemoglobin and haematocrit with 0.68 [13]. Similarly, Ryan et al. found that initial haematocrit predicted mortality with AUC values ranging from 0.60–0.73 depending on injury severity [52], while Thorson et al. demonstrated that admission haematocrit < 33% was associated with increased transfusion requirements and mortality [49]. Mathais et al. found that prehospital haemoglobin predicted need for transfusion with AUC 0.82 using a threshold of 11.45 g/dL, higher than our admission threshold of 10.45 g/dL [53]. However, these studies examined only admission values, whereas our serial measurements reveal that predictive performance varies substantially over time, with AUC values remaining consistently poor (0.44–0.68) across all time points and never exceeding the threshold for acceptable discrimination (AUC > 0.70). The clinical utility of any biomarker threshold depends not only on its AUC but also on whether it provides actionable information beyond readily available clinical assessment. Given that injury severity scores (ISS, APACHE II) and physiological parameters (blood pressure, lactate) are routinely available and demonstrated strong associations with mortality in our cohort (Table 1), the incremental predictive value of serial haemoglobin and haematocrit measurements beyond these established markers remains uncertain and warrants further investigation.

While crude comparisons demonstrated differences in haemoglobin and haematocrit between survivors and non-survivors, these associations did not survive adjustment for multiple testing in regression models. This pattern—significant group differences but non-significant regression coefficients after FDR correction—suggests that the observed haematological differences reflect confounding by injury severity and resuscitation intensity rather than independent prognostic relationships. The failure of any timepoint to demonstrate independent predictive value after controlling for established trauma severity markers (ISS, APACHE II) and resuscitation volumes indicates that serial haemoglobin and haematocrit measurements provide limited incremental prognostic information beyond readily available clinical parameters.

Several important limitations must be acknowledged. An important limitation is the 26-year study period (1996–2022), which spans major paradigm shifts in trauma resuscitation. Early in the study period, large-volume crystalloid resuscitation was standard practice, whereas damage control resuscitation with balanced blood product transfusion (1:1:1 ratios) and restrictive crystalloid use became standard after approximately 2010 [2,12]. These temporal changes in resuscitation strategy likely influenced the relationship between haemoglobin/haematocrit and mortality, as aggressive crystalloid administration produces dose-dependent haemodilution while early blood product transfusion transiently elevates haematological parameters [54,55]. The thresholds we identified may therefore represent an average across incompatible resuscitation eras and may not reflect current practice. Ideally, this analysis would be stratified by time period (e.g., 1996–2008 vs. 2009–2022) to account for these temporal changes, though sample size limitations precluded such stratification. A fundamental limitation of this analysis is the inability to fully disentangle endogenous haematological deterioration from iatrogenic haemodilution caused by resuscitation. The haemoglobin and haematocrit values measured at each time point represent the sum of haemorrhage-driven decline, endogenous compensatory mechanisms, and resuscitation-induced modification. Non-survivors received significantly greater volumes of intravenous fluids and erythrocyte concentrates throughout the observation period, meaning their haemoglobin and haematocrit trajectories were subject to systematically different levels of iatrogenic modification compared to survivors. Each 500 mL of crystalloid administration produces measurable haemodilution with decreased haemoglobin and haematocrit [54,55], and aggressive crystalloid resuscitation is associated with worsened coagulopathy, increased acute respiratory distress syndrome risk, and potentially worse outcomes [3,56]. The thresholds we identified may therefore operationally identify ‘patients who received aggressive resuscitation’ as much as ‘patients with intrinsically compromised haematological reserve.’ While we adjusted for cumulative resuscitation volumes in multivariable models, residual confounding likely persists because resuscitation decisions themselves are driven by clinical assessment of haemorrhage severity, creating complex bidirectional relationships between haemoglobin values, transfusion decisions, and outcomes. We did not perform stratified analyses by resuscitation intensity (e.g., high vs. low transfusion volume) to determine whether the observed Hb/Hct-mortality associations reflect endogenous physiological deterioration or iatrogenic modification from resuscitation. Such stratification would help clarify whether our thresholds identify patients with intrinsically compromised haematological reserve versus those who received aggressive resuscitation, but was beyond the scope of this retrospective analysis. The reported thresholds should therefore be considered hypothesis-generating observations that require prospective validation in cohorts with standardised resuscitation protocols before clinical implementation.

Second, data quality issues at the 6-h time point for male haemoglobin (acknowledged data entry errors) limit interpretation of findings at this interval. The analysis was therefore rerun without the erroneous values, and the findings reported accordingly.

Third, this single-centre study from a Level 1 trauma centre may not generalise to other settings with different patient populations, injury patterns, or resuscitation protocols. The unequal sex distribution (73.5% male) may limit statistical power for detecting sex-specific effects, particularly in subgroup analyses.

Fourth, we lacked data on hormonal status (menstrual cycle phase, menopausal status, oral contraceptive use, hormone replacement therapy) that critically modulates trauma outcomes in females [39,46]. The retrospective design precluded standardisation of blood sampling times, meaning exact time points cannot be guaranteed.

Fifth, we did not account for baseline pre-injury haemoglobin and haematocrit values, limiting our ability to distinguish acute haemorrhage-related changes from pre-existing anaemia or polycythaemia. Finally, unmeasured confounders including extent of ongoing haemorrhage, surgical interventions, and comorbidities may influence the observed associations.

## 5. Conclusions

In this retrospective cohort of 3059 polytrauma patients, haemoglobin and haematocrit demonstrated time-sensitive associations with early death within 72 h, with sex-specific temporal patterns suggesting differential physiological responses to haemorrhage. However, these relationships are substantially confounded by resuscitation practices, as non-survivors received significantly greater fluid and transfusion volumes that independently affect haematological parameters through haemodilution [12,55]. After adjusting for injury severity, physiological status, and resuscitation volumes in multivariable models, Haemoglobin and Haematocrit at 2 h for males remain significant before the Benjamini-Hochberg correction. After the correction, no values remain significant.

The sex-specific differences we observed—particularly the early predictive window in females versus the more distributed pattern in males—align with established sex dimorphisms in coagulation, hormonal modulation of haemorrhagic response, and inflammatory signalling [32,33,34,35,36,37,38,40,41,42,43,44,45,46,57]. However, the clinical utility of the specific thresholds identified (haemoglobin nadir at 2 h in females, 48 h in males; haematocrit nadir at 1 h in females, 3 h in males) remains uncertain given the inability to distinguish endogenous physiological changes from iatrogenic modification. These findings should be considered hypothesis-generating and require prospective validation in cohorts with standardised resuscitation protocols before informing clinical decision-making regarding blood product administration or fluid resuscitation strategies [26].

## Figures and Tables

**Table 1:** Baseline characteristics of the overall patient sample analysing the complete cohort as well as the cohort split up according to their early death within 72 h. The statistical significance was analysed using the Mann-Whitney U Test. Age in years, BMI [kg/m^2^], Temperature [°C], Blood pressure [mmHg], CRP [mg/L], PCT [ng/mL], and lactate [mmol/L].

Baseline Characteristics	Overall Patient Sample N = 3059	Patients with Early Death Within 72 h N = 934	Patients Without Early Death Within 72 h N = 2125	*p*-Value
Age (Mean, SD)	45.81 ± 20.13	52.15 ± 22.26	43.02 ± 18.45	<0.001
Male	73.55%, N = 2250	72.91%, N = 681	73.84%, N = 1569	-
Female	26.25%, N = 809	31.27%, N = 253	68.73%, N = 556	-
Early Death within 72 h	23.11%, N = 707	75.7%, N = 707	-	-
Blunt Trauma	91.83%, N = 2809	89.4%, N = 835	92.94%, N = 1975	-
Head Injury	40.41%, N = 1236	38.12%, N = 356	41.41%, N = 880	-
Body Mass Index (BMI) at admission (Mean, SD)	24. 98 ± 4.28	25.49 ± 4.89	24.88 ± 4.15	0.017
ISS (Median, IQR)	27 (11–43)	34 (9–59)	26 (12–40)	<0.001
NISS (Median, IQR)	41 (18–64)	50 (25–75)	34 (18–50)	<0.001
APACHE II at admission (Median, IQR)	15 (2–28)	22 (7.68–29.68)	11 (0–23)	<0.001
GCS at admission (Median, IQR)	7 (3–15)	3 (3–15)	13 (3–15)	<0.001
Temperature at admission (Mean, SD)	35.47 ± 1.78	34.79 ± 2.38	35.72 ± 1.42	<0.001
Systolic Blood Pressure at admission (Mean, SD)	127.67 ± 31.12	122.43 ± 34.66	123.41 ± 25.18	0.022
C reactive protein (CRP) at admission (Mean, SD)	14.11 ± 41.34	10.7 ± 34.19	15.44 ± 43.78	0.712
pH at admission (Mean, SD)	7.31 ± 0.13	7.26 ± 0.17	7.33 ± 0.11	<0.001
Procalcitonin (PCT) at admission (Mean, SD)	1.39 ± 4.72	0.08 ± 0.03	1.48 ± 4.86	0.136
Lactate at admission (Mean, SD)	3.03 ± 2.61	4.18 ± 3.51	2.55 ± 1.93	<0.001

**Table 2:** Baseline characteristics of the overall patient sample regarding haemoglobin (g/dL) and okokhaematocrit (%), showing N, the median and IQR (Interquartile range).

	N	Mean, 95% Confidence Interval
Haematocrit at admission	2716	30.66 (30.08–31.25)
Haematocrit 1 h	980	28.42 (27.44–29.41)
Haematocrit 2 h	1008	27.13 (26.34–27.92)
Haematocrit 3 h	982	27.58 (26.81–28.35)
Haematocrit 4 h	1086	28.20 (27.51–28.88)
Haematocrit 6 h	1147	28.36 (27.72–29.01)
Haematocrit 8 h	1084	28.34 (27.71–28.96)
Haematocrit 12 h	1352	28.96 (28.47–29.46)
Haematocrit 24 h	1708	28.19 (27.79–28.59)
Haematocrit 48 h	1452	27.67 (27.19–28.13)
Haemoglobin at admission	2605	11.19 (11.08–11.30)
Haemoglobin 1 h	944	10.13 (9.94–10.32)
Haemoglobin 2 h	937	9.63 (9.47–9.80)
Haemoglobin 3 h	883	9.36 (9.48–9.78)
Haemoglobin 4 h	869	9.81 (9.66–9.96)
Haemoglobin 6 h	870	9.72 (9.58–9.87)
Haemoglobin 8 h	701	9.67 (9.53–9.85)
Haemoglobin 12 h	1035	9.94 (9.82–10.1)
Haemoglobin 24 h	1611	9.81 (9.72–9.89)
Haemoglobin 48 h	1381	9.51 (9.42–9.59)

**Table 3:** Baseline characteristics of the overall patient sample regarding volume substitution (ml) and number of erythrocyte concentrates (EC), showing N, the median and IQR (Interquartile range) in males and females.

	Median (IQR), N in Females	Median (IQR), N in Males
Volume at admission	933.61 (858.18–1009.05), N = 576	1209.72 (1141.49–1277.96), N = 1628
Volume 1 h	2113.37 (1948.29–2278.45), N = 627	2392.85 (2287.12–2498.57), N = 1757
Volume 2 h	2706.71 (2483.22–2930.21), N = 526	3303.45 (3143.11–3463.80), N = 526
Volume 3 h	3349.02 (3082.67–3615.37), N = 516	4023.59 (3831.14–4216.04), N = 1382
Volume 4 h	3853.28 (3554.64–4151.91), N = 513	4696.29 (4468.03–4924.54), N = 1364
Volume 6 h	4607.07 (4264.39–4949.75), N = 510	5501.22 (5267.63–5734.82), N = 1355
Volume 8 h	5107.19 (4749.49–5464.90), N = 494	6274.11 (6017.10–6531.12), N = 1333
Volume 12 h	6353.01 (5944.20–6761.81), N = 575	7471.21 (7194.97–7747.44), N = 1615
Volume 24 h	8504.87 (7991.83–9017.90), N = 561	10,016.97 (9656.75–10,377.19), N = 1524
Volume 48 h	11,845.20 (11,017.91–12,672.48), N = 502	13,718.71 (13,311.91–14,125.51), N = 1435
EC Concentrate at admission	0.02 (0.00–0.04), N = 725	0.06 (0.03–0.09), N = 2032
EC Concentrate 1 h	0.54 (0.38–0.70), N = 729	0.51 (0.42–0.60), N = 2043
EC Concentrate 2 h	1.145 (0.89–1.39), N = 737	1.182 (1.02–1.35), N = 2048
EC Concentrate 3 h	1.63 (1.3–1.96), N = 735	1.74 (1.52–1.96), N = 2043
EC Concentrate 4 h	1.99 (1.61–2.37), N = 735	2.09 (1.83–2.34), N = 2042
EC Concentrate 6 h	2.47 (2.01–2.93), N = 738	2.53 (2.23–2.83), N = 2045
EC Concentrate 8 h	2.62 (2.13–3.11), N = 738	2.79 (2.47–3.10), N = 2035
EC Concentrate 12 h	3.14 (2.56–3.72), N = 734	3.33 (2.98–3.69), N = 2037
EC Concentrate 24 h	3.49 (2.87–4.11), N = 739	3.82 (3.42–4.22), N = 2036
EC Concentrate 48 h	3.86 (3.20–4.51), N = 741	4.37 (3.88–4.85), N = 2041

**Table 4:** Early death within 72 h in Males and Females regarding volume substitution (ml) and number of erythrocyte concentrates (EC), showing N, the median and IQR (Interquartile range).

	Median (IQR), N in Females with Early Death Within 72 h	Median (IQR), N in Females Without Early Death Within 72 h	Median (IQR), N in Males with Early Death Within 72 h	Median (IQR), N in Males Without Early Death Within 72 h
Volume at admission	1042.76 (890.12–1195.39), N = 156	893.07 (806.46–979.68), N = 420	1392.12 (1244.97–1539.28), N = 433	1143.63 (1067.69–1219.58), N = 1195
Volume 1 h	2712.51 (2272.14–3152.88), N = 166	1897.63 (1742.00–2053.26), N = 461	2927.33 (2670.25–3184.22), N = 446	2211.05 (2101.04–2321.06), N = 1311
Volume 2 h	3259.10 (2610.31–3907.89), N = 130	2525.38 (2319.31–2731.44), N = 396	3956.42 (3548.79–4364.05), N = 314	3117.40 (2948.44–3286.36), N = 1102
Volume 3 h	3897.11 (3111.38–4682.83), N = 121	3181.12 (2929.44–3431.80), N = 195	4611.52 (4111.22–5111.81), N = 283	3872.19 (3667.83–4076.56), N = 1099
Volume 4 h	4518.71 (3636.11–5401.31), N = 120	3650.09 (3368.41–3931.78), N = 393	5510.15 (4836.58–6183.72), N = 268	4497.28 (4266.69–4727.86), N = 1096
Volume 6 h	5135.95 (4137.04–6134.86), N = 116	4451.37 (4117.58–4785.15), N = 394	6218.63 (5472.47–6964.80), N = 260	5330.88 (5102.84–5558.92), N = 1095
Volume 8 h	5408.18 (4326.68–6489.68), N = 107	5023.98 (4675.79–5372.16), N = 387	6862.02 (6057.81–7666.24), N = 241	6144.36 (5885.52–6403.21), N = 1092
Volume 12 h	6656.30 (5323.72–7988.89), N = 119	6273.86 (5889.77–6657.94), N = 456	7832.83 (7074.04–8591.63), N = 309	7385.64 (7094.66–7676.63), N = 1306
Volume 24 h	8564.58 (6719.00–10,410.16), N = 106	8490.96 (8021.48–8960.44), N = 455	10,239.99 (9248.97–11,231.00), N = 258	9972.16 (9587.51–10,356.81), N = 1284
Volume 48 h	11,173.07 (8509.42–13,836.72), N = 88	11,988.06 (11,153.84–12,822.28), N = 414	13,939.94 (12,700.72–15,179.17), N = 229	13,676.70 (13,252.82–14,100.57), N = 1206
EC Concentrate at admission	0.02 (0.01–0.05), N = 223	0.02 (0.00–0.04), N = 502	0.07 (0.01–0.13), N = 610	0.05 (0.02–0.09), N = 1422
EC Concentrate 1 h	1.07 (0.66–1.47), N = 226	0.30 (0.18–0.43), N = 503	0.99 (0.75–1.23), N = 615	0.03 (0.22–0.38), N = 1428
EC Concentrate 2 h	1.98 (1.37–2.59), N = 229	0.77 (0.54–0.99), N = 508	1.904 (1.493–2.315), N = 614	0.873 (0.723–1.023), N = 1434
EC Concentrate 3 h	2.63 (1.83–3.43), N = 225	1.19 (0.88–1.50), N = 510	2.59 (2.04–3.14), N = 607	1.83 (1.18–1.59), N = 1436
EC Concentrate 4 h	3.02 (2.09–3.96), N = 225	1.53 (1.17–1.89), N = 510	2.95 (2.33–3.57), N = 607	1.72 (1.47–1.97), N = 1435
EC Concentrate 6 h	3.57 (2.45–4.68), N = 227	1.98 (1.55–2.42), N = 511	3.43 (2.72–4.15), N = 607	2.15 (1.85–2.44), N = 1438
EC Concentrate 8 h	3.63 (2.45–4.81), N = 225	2.17 (1.70–2.65), N = 513	3.61 (2.86–4.36), N = 598	2.45 (2.13–2.77), N = 1437
EC Concentrate 12 h	4.13 (2.76–5.49), N = 224	2.70 (2.12–3.29), N = 510	4.25 (3.39–5.10), N = 598	2.95 (2.60–3.31), N = 1439
EC Concentrate 24 h	4.47 (3.02–5.92), N = 224	3.06 (2.43–3.69), N = 515	4.62 (3.70–5.54), N = 597	3.49 (3.08–3.90), N = 1439
EC Concentrate 48 h	4.60 (3.11–6.09), N = 223	3.53 (2.85–4.22), N = 518	4.76 (3.84–5.68), N = 595	4.21 (3.64–4.78)

**Table 5:** Univariable and multivariable associations between haemoglobin (g/dL), haematocrit (%) and early death within 72 h by sex and time point.

Time After Admission	Sex	Predictor	N	Crude OR (95% CI)	*p*-Value Multivariable Binary Logistic	Adjusted OR (95%)	Nagelkerke R^2^
0 h	Male	Haemoglobin	1934	0.92 (0.67–1.27)	0.702	1.00 (0.96–1.05)	0.39
0 h	Male	Haematocrit	2020	0.98 (0.92–1.04)	0.881	0.99 (0.98–1.02)	0.39
0 h	Female	Haemoglobin	672	Not estimable †	0.708	1.01 (0.97–1.05)	0.39
0 h	Female	Haematocrit	696	0.94 (0.79–1.11)	0.785	1.01 (0.97–1.05)	0.39
1 h	Male	Haemoglobin	698	0.79 (0.49–1.29)	0.988	1.00 (0.92–1.09)	0.29
1 h	Male	Haematocrit	729	0.98 (0.90–1.07)	0.825	0.99 (0.97–1.03)	0.28
1 h	Female	Haemoglobin	246	Not estimable †	0.475	0.95 (0.81–1.10)	0.33
1 h	Female	Haematocrit	251	1.04 (0.83–1.31)	0.613	0.99 (0.94–1.04)	0.31
2 h	Male	Haemoglobin	699	2.32 (1.19–4.53)	0.01	1.13 (1.03–1.24)	0.24
2 h	Male	Haematocrit	758	1.05 (0.96–1.16)	0.009	1.04 (1.01–1.07)	0.23
2 h	Female	Haemoglobin	238	Not estimable †	0.270	1.12 (0.91–1.38)	0.23
2 h	Female	Haematocrit	250	0.96 (0.75–1.21)	0.225	1.02 (0.91–1.11)	0.23
3 h	Male	Haemoglobin	660	0.51 (0.25–1.04)	0.710	1.02 (0.91–1.15)	0.25
3 h	Male	Haematocrit	740	1.06 (0.95–1.18)	0.576	1.01 (0.97–1.05)	0.21
3 h	Female	Haemoglobin	238	Not estimable †	0.541	1.05 (0.86–1.27)	0.31
3 h	Female	Haematocrit	242	1.32 (0.96–1.82)	0.859	1.01 (0.95–1.07)	0.28
4 h	Male	Haemoglobin	648	1.19 (0.66–2.16)	0.775	1.01 (0.94–1.08)	0.22
4 h	Male	Haematocrit	820	0.89 (0.79–0.99)	0.967	0.99 (0.97–1.03)	0.19
4 h	Female	Haemoglobin	223	Not estimable †	0.794	0.98 (0.80–1.19)	0.34
4 h	Female	Haematocrit	266	0.97 (0.75–1.26)	0.32	1.04 (0.97–1.11)	0.25
6 h	Male	Haemoglobin	646	1.85 (1.07–3.22)	0.873	1.00 (0.99–1.00)	0.17
6 h	Male	Haematocrit	855	1.17 (1.03–1.32)	0.454	1.01 (0.98–1.05)	0.16
6 h	Female	Haemoglobin	223	Not estimable †	0.292	0.89 (0.71–1.11)	0.27
6 h	Female	Haematocrit	292	0.98 (0.80–1.21)	0.811	0.99 (0.93–1.06)	0.21
8 h	Male	Haemoglobin	519	0.61 (0.31–1.22)	0.316	0.93 (0.80–1.07)	0.22
8 h	Male	Haematocrit	814	0.88 (0.78–0.99)	0.640	1.01 (0.97–1.05)	0.15
8 h	Female	Haemoglobin	182	Not estimable †	0.973	1.01 (0.76–1.32)	0.32
8 h	Female	Haematocrit	270	0.98 (0.67–1.44)	0.876	0.99 (0.92–1.07)	0.23
12 h	Male	Haemoglobin	740	0.89 (0.48–1.67)	0.321	0.94 (0.82–1.07)	0.18
12 h	Male	Haematocrit	994	1.14 (0.99–1.30)	0.923	1.00 (0.96–1.04)	0.16
12 h	Female	Haemoglobin	297	Not estimable †	0.833	1.03 (0.80–1.31)	0.17
12 h	Female	Haematocrit	358	0.82 (0.63–1.08)	0.906	0.99 (0.93–1.07)	0.19
24 h	Male	Haemoglobin	1218	1.02 (0.44–2.36)	0.606	0.97 (0.88–1.08)	0.17
24 h	Male	Haematocrit	1285	0.83 (0.71–0.97)	0.799	0.99 (0.96–1.03)	0.16
24 h	Female	Haemoglobin	366	Not estimable †	0.824	1.03 (0.80–1.33)	0.17
24 h	Female	Haematocrit	423	0.71 (0.49–1.03)	0.931	0.99 (0.92–1.08)	0.14
48 h	Male	Haemoglobin	1047	1.11 (0.50–2.50)	0.625	0.98 (0.91–1.06)	0.15
48 h	Male	Haematocrit	1099	1.09 (0.94–1.26)	0.619	1.01 (0.96–1.06)	0.14
48 h	Female	Haemoglobin	336	Not estimable †	0.821	1.01 (0.94–1.08)	0.14
48 h	Female	Haematocrit	353	1.54 (1.01–2.35)	0.287	1.05 (0.96–1.15)	0.11

Crude (univariable) and adjusted (multivariable) odds ratios with 95% confidence intervals for the association between haemoglobin (g/dL) and haematocrit (%) and early death within 72 h, stratified by sex and time point. Multivariable models adjusted for Injury Severity Score (ISS), age, APACHE II score at admission, cumulative fluid volume administered, and cumulative erythrocyte concentrate units transfused up to each time point. Nagelkerke R^2^ indicates model fit. N = number of patients with complete data for all covariates at each time point. OR = odds ratio; CI = confidence interval. † Odds ratio could not be reliably estimated due to quasi-complete separation or model convergence failure, likely reflecting small sample size and low event rates in this subgroup. *p*-values from Mann-Whitney U tests, univariable binary logistic regression, and multivariable binary logistic regression (adjusted for ISS, age, APACHE II, cumulative fluid volume, and cumulative erythrocyte concentrate transfusion) are shown before and after Benjamini-Hochberg false discovery rate (FDR) correction for 40 comparisons per test type. Significance threshold: adjusted *p* < 0.05.

**Table 6:** Comparison of crude and FDR-corrected associations between serial haemoglobin and haematocrit measurements and early death within 72 h, stratified by sex.

Gender	Predictor	Time After Admission	*p*-Value (from Mann-Whitney U)	Adjusted *p*-Value	Significant?	*p*-Value Univariable Binary log. Regression	Adjusted *p*-Value	Significant?	*p*-Value Multivariable Binary log. Regression	Adjusted *p*-Value	Significant?
Male	Haemoglobin	0 h	<0.001	<0.001	YES	0.604	1	NO	0.702	0.980	NO
Male	Haemoglobin	1 h	<0.001	<0.001	YES	0.348	0.819	NO	0.988	0.988	NO
Male	Haemoglobin	2 h	0.740	0.800	NO	0.013	0.260	NO	0.01	0.200	NO
Male	Haemoglobin	3 h	0.044	0.135	NO	0.065	0.289	NO	0.710	0.980	NO
Male	Haemoglobin	4 h	0.169	0.294	NO	0.566	1	NO	0.775	0.980	NO
Male	Haemoglobin	6 h	0.093	0.233	NO	0.029	0.263	NO	0.873	0.980	NO
Male	Haemoglobin	8 h	0.131	0.262	NO	0.162	0.499	NO	0.318	0.980	NO
Male	Haemoglobin	12 h	0.003	0.013	YES	0.724	1	NO	0.321	0.980	NO
Male	Haemoglobin	24 h	0.031	0.103	NO	0.970	1	NO	0.606	0.980	NO
Male	Haemoglobin	48 h	0.227	0.337	NO	0.796	1	NO	0.625	0.980	NO
Male	Haematocrit	0 h	<0.001	<0.001	YES	0.427	0.918	NO	0.881	0.980	NO
Male	Haematocrit	1 h	<0.001	<0.001	YES	0.613	1	NO	0.825	0.980	NO
Male	Haematocrit	2 h	0.654	0.769	NO	0.298	0.745	NO	0.009	0.200	NO
Male	Haematocrit	3 h	0.073	0.209	NO	0.279	0.744	NO	0.576	0.980	NO
Male	Haematocrit	4 h	0.086	0.229	NO	0.046	0.263	NO	0.967	0.988	NO
Male	Haematocrit	6 h	0.28	0.396	NO	0.013	0.260	NO	0.454	0.980	NO
Male	Haematocrit	8 h	0.116	0.258	NO	0.033	0.263	NO	0.640	0.980	NO
Male	Haematocrit	12 h	0.009	0.033	YES	0.059	0.289	NO	0.923	0.980	NO
Male	Haematocrit	24 h	0.005	0.020	YES	0.025	0.263	NO	0.799	0.980	NO
Male	Haematocrit	48 h	0.217	0.333	NO	0.245	0.700	NO	0.619	0.980	NO
Female	Haemoglobin	0 h	<0.001	<0.001	YES	0.997	1	NO	0.708	0.980	NO
Female	Haemoglobin	1 h	0.003	0.013	YES	0.998	1	NO	0.475	0.980	NO
Female	Haemoglobin	2 h	0.574	0.696	NO	0.999	1	NO	0.270	0.980	NO
Female	Haemoglobin	3 h	0.693	0.792	NO	0.998	1	NO	0.541	0.980	NO
Female	Haemoglobin	4 h	0.722	0.800	NO	1	1	NO	0.794	0.980	NO
Female	Haemoglobin	6 h	0.287	0.396	NO	1	1	NO	0.292	0.980	NO
Female	Haemoglobin	8 h	0.168	0.294	NO	0.999	1	NO	0.316	0.980	NO
Female	Haemoglobin	12 h	0.100	0.235	NO	0.999	1	NO	0.833	0.980	NO
Female	Haemoglobin	24 h	0.909	0.915	NO	0.999	1	NO	0.824	0.980	NO
Female	Haemoglobin	48 h	0.466	0.5825	NO	0.999	1	NO	0.821	0.980	NO
Female	Haematocrit	0 h	<0.001	<0.001	YES	0.436	0.918	NO	0.785	0.980	NO
Female	Haematocrit	1 h	<0.001	<0.001	YES	0.741	1	NO	0.613	0.980	NO
Female	Haematocrit	2 h	0.183	0.305	NO	0.708	1	NO	0.225	0.980	NO
Female	Haematocrit	3 h	0.915	0.915	NO	0.085	0.309	NO	0.859	0.980	NO
Female	Haematocrit	4 h	0.214	0.333	NO	0.808	1	NO	0.320	0.980	NO
Female	Haematocrit	6 h	0.127	0.262	NO	0.878	1	NO	0.811	0.980	NO
Female	Haematocrit	8 h	0.160	0.294	NO	0.932	1	NO	0.876	0.980	NO
Female	Haematocrit	12 h	0.389	0.502	NO	0.159	0.498	NO	0.906	0.980	NO
Female	Haematocrit	24 h	0.789	0.831	NO	0.074	0.296	NO	0.931	0.980	NO
Female	Haematocrit	48 h	0.329	0.439	NO	0.045	0.263	NO	0.287	0.980	NO

**Table 7:** AUC and 95% CI in Males and Females regarding Haemoglobin (g/dL) and Haematocrit (%).

Time After Admission	Sex	Predictor	AUC (95% CI)
0 h	Male	Haemoglobin	0.51 (0.33–0.68)
0 h	Male	Haematocrit	0.47 (0.37–0.57)
0 h	Female	Haemoglobin	0.65 (0.44–0.86)
0 h	Female	Haematocrit	0.47 (0.27–0.67)
1 h	Male	Haemoglobin	0.57 (0.41–0.72)
1 h	Male	Haematocrit	0.51 (0.41–0.60)
1 h	Female	Haemoglobin	0.44 (0.16–0.72)
1 h	Female	Haematocrit	0.55 (0.36–0.74)
2 h	Male	Haemoglobin	0.63 (0.48–0.78)
2 h	Male	Haematocrit	0.51 (0.40–0.62)
2 h	Female	Haemoglobin	0.61 (0.27–0.95)
2 h	Female	Haematocrit	0.60 (0.39–0.81)
3 h	Male	Haemoglobin	0.55 (0.39–0.71)
3 h	Male	Haematocrit	0.54 (0.45–0.63)
3 h	Female	Haemoglobin	0.68 (0.44–0.93)
3 h	Female	Haematocrit	0.68 (0.53–0.82)
4 h	Male	Haemoglobin	0.55 (0.39–0.71)
4 h	Male	Haematocrit	0.47 (0.37–0.58)
4 h	Female	Haemoglobin	0.67 (0.45–0.88)
4 h	Female	Haematocrit	0.65 (0.46–0.83)
6 h	Male	Haemoglobin	0.63 (0.47–0.78)
6 h	Male	Haematocrit	0.55 (0.45–0.64)
6 h	Female	Haemoglobin	0.52 (0.30–0.75)
6 h	Female	Haematocrit	0.50 (0.32–0.68)
8 h	Male	Haemoglobin	0.46 (0.29–0.62)
8 h	Male	Haematocrit	0.45 (0.34–0.56)
8 h	Female	Haemoglobin	0.31 (0.05–0.67)
8 h	Female	Haematocrit	0.51 (0.35–0.67)
12 h	Male	Haemoglobin	0.53 (0.37–0.68)
12 h	Male	Haematocrit	0.52 (0.43–0.62)
12 h	Female	Haemoglobin	0.49 (0.28–0.70)
12 h	Female	Haematocrit	0.40 (0.23–0.56)
24 h	Male	Haemoglobin	0.57 (0.42–0.72)
24 h	Male	Haematocrit	0.48 (0.39–0.57)
24 h	Female	Haemoglobin	0.52 (0.14–0.89)
24 h	Female	Haematocrit	0.44 (0.25–0.63)
48 h	Male	Haemoglobin	0.59 (0.43–0.75)
48 h	Male	Haematocrit	0.54 (0.45–0.63)
48 h	Female	Haemoglobin	0.65 (0.39–0.01)
48 h	Female	Haematocrit	0.66 (0.51–0.80)

## Data Availability

The data presented in this study are available on request from the corresponding author. (Reason for restriction, the data are not publicly available due to privacy restrictions.).
